# Rotavirus Double Infection Model to Study Preventive Dietary Interventions

**DOI:** 10.3390/nu11010131

**Published:** 2019-01-10

**Authors:** Maria del Mar Rigo-Adrover, Karen Knipping, Johan Garssen, Sandra Saldaña-Ruíz, Àngels Franch, Margarida Castell, Francisco J Pérez-Cano

**Affiliations:** 1 Departament de Bioquímica i Fisiologia, Facultat de Farmàcia i Ciències de l’Alimentació, University of Barcelona (UB), Av. Joan XXIII 27-31, 08028 Barcelona, Spain; mmrigo@gmail.com (M.d.M.R.-A.); ssaldana1@hotmail.com (S.S.-R.); angelsfranch@ub.edu (À.F.); margaridacastell@ub.edu (M.C.); 2 Institut de Recerca en Nutrició i Seguretat Alimentària (INSA), C/ Prat de la Riba 171, 08921 Santa Coloma de Gramanet, 08921 Barcelona, Spain; 3 Nutricia Research, 3584 CT Utrecht, The Netherlands; Karen.Knipping@danone.com (K.K.); j.garssen@uu.nl (J.G.); 4 Division of Pharmacology, Utrecht Institute for Pharmaceutical Sciences, Faculty of Science, Utrecht University, 3584 CA Utrecht, The Netherlands

**Keywords:** rotavirus, diarrhea, rat, model, double-infection

## Abstract

Rotaviruses are the main cause of acute diarrhea among young children worldwide with an increased frequency of reinfection. Several life style factors, such as dietary components, may influence such processes by affecting the outcome of the first rotavirus infection and therefore having a beneficial impact on the anti-rotavirus immune responses during any subsequent reinfections. The aim of this research was to develop a double-infection model in rat that mimics real-life clinical scenarios and would be useful in testing whether nutritional compounds can modulate the rotavirus-associated disease and immune response. Three experimental designs and a preventive dietary-like intervention were conducted in order to achieve a differential response in the double-infected animals compared to the single-infected ones and to study the potential action of a modulatory agent in early life. Diarrhea was only observed after the first infection, with a reduction of fecal pH and fever. After the second infection an increase in body temperature was also found. The immune response against the second infection was regulated by the preventive effect of the dietary-like intervention during the first infection in terms of specific antibodies and DTH. A rotavirus-double-infection rat model has been developed and is suitable for use in future preventive dietary intervention studies.

## 1. Introduction

Diarrhea is the fourth highest cause of mortality among children under the age of five worldwide [1], and rotavirus is the aetiological agent responsible for 37% of these deaths [2]. Almost every child in the world will be infected with rotavirus at some time in their first 3 years of life [3], however, it can also infect adults [4], and the presence of rotavirus particles in extra-intestinal tissues has also been reported [5,6,7,8]. Rotavirus belongs to the family Reoviridae, which are non-enveloped, icosahedral, double-stranded RNA viruses covered by triple-layer capsids. The viral genome encodes for six structural proteins and six non-structural proteins [6,9,10,11]. Group A rotavirus are the main human pathogens and they are transmitted via the fecal—oral route, with a higher prevalence in winter. They infect the mature absorptive enterocytes of the small intestine, although the exact entry mechanism is still unknown [12]. In children the main symptoms are fever, vomiting, abdominal cramps and diarrhea, lasting for 3 to 8 days [6,13], and the virus can be spread from 2 days before and up to 8 days after the onset of diarrhea. Oral rehydration is the most usual treatment [14,15]. 

Immunity after a rotavirus infection is incomplete and several reinfections usually occur, which tend to be less severe than the first [13]. Innate and adaptive immune responses are induced by rotavirus infections, including cytokine and specific antibody (Ab) production [16,17]. The initial immune response is the presentation of antigen to T and B lymphocytes through macrophages and dendritic cells (DC). DC seem to be crucial for cellular response activation and represent a link between innate and adaptive immunity [18]. Natural killer (NK) lymphocytes constitute the first line of defense against the virus and destroy infected cells. T cells also lyse infected cells and produce cytokines. Finally, B cells produce Ab both locally and systemically, and are required for long-term protection [19]. Protection against rotavirus seems to be positively correlated with immunoglobulin (Ig) A [20,21], although IgG and IgM also confer some protection [22].

The introduction of two oral vaccines against rotavirus into routine vaccination programs (RotaTeq, Merck & Co, White House Station, NJ, USA) and Rotarix, GSK Biologicals (Rixensart, Belgium) has shown a reduction in the health burden of severe childhood diarrhea [2,9], but their implementation is still not as widespread as had been expected, due among other reasons, mainly their cost and the need for refrigerated storage [3,15]. Thus, it is necessary to develop alternative approaches to control the rotavirus disease.

Interventional nutrition studies in humans, and particularly in infants, present certain difficulties. For this reason, several animal models have been used to better understand rotavirus pathology and infection, and also to study the vaccine efficacy or the amelioration of the disease course through dietary intervention in early life with bioactive compounds (such as whey proteins, prebiotics and probiotics) [23,24,25,26]. Most of the existing studies in this subject have focused on the evaluation of clinical markers, such as the incidence and severity of diarrhea, while others have also studied the presence of the virus in serum, tissues and/or feces, and evaluated the immune function through evaluating Ab titers, among others [25]. In the rotavirus model context, all models add valuable information, but large animals (such as cows, pigs and sheep) have been used as experimental subjects; however, these studies involve high costs and sometimes also require long periods of study. In contrast, mice models of rotavirus infection also exist and have been very helpful in the context of the mechanisms of viral adhesion, replication and diarrhea induction because severe diarrhea can usually be obtained after rotavirus inoculation of suckling mice. However, this success in diarrhea induction makes the observation of the benefits of certain nutritional interventions difficult, because the intervention with dietary components at physiological doses is not strong enough to ameliorate the process. This fact is described as a current limitation in immunonutrition studies [27]. However, the neonatal rat model, with a moderate severity of disease [28,29] and susceptible to heterologous rotavirus infection, has been demonstrated as being suitable for immunonutrition studies, providing substantial scientific evidence as well as having a cost-effective ratio [24].

Most rotavirus animal models include just one rotavirus infection (i.e., single-infection models), but due to the frequency of reinfection in human infants, a double-infection model would provide additional information. At present, rotavirus double-infection models already exist for some species [30,31,32,33,34], but there is no rat model. In the double-infection models, it would be interesting to evaluate the effect of two different rotavirus because humans are usually infected by different strains of rotavirus during their lifetime. In addition, it would be interesting to fully evaluate the differential response of both infections together compared to a single infection response, and how protection by nutritional agents against the first infection, i.e., by a preventive intervention, will affect the second infection, mainly in terms of immune response. Therefore, the aim of the present study was to develop and characterize a neonatal rat double rotavirus infection model that would be suitable for use in future dietary interventional studies.

## 2. Materials and Methods

### 2.1. Animals

G14 pregnant Lewis rats were obtained from Harlan (Barcelona, Spain) and Janvier Labs (La Plaine Saint Denis Cedex, France). They were housed in individual cages, monitored daily and allowed to deliver at term. The day of birth was registered as day 1 of life. The pups had free access to the nipples and rat diet. The animals were housed under controlled temperature and humidity conditions, in a 12:12 h light/dark cycle. They were located in a special safe isolated room at the Animal Service of the Faculty of Pharmacy, University of Barcelona (UB), designed and authorized for working under biosecurity level 2 conditions. Dams were fed with a commercial diet corresponding to the American Institute of Nutrition 93M formulation and given water ad libitum. The rotavirus were intragastrically inoculated as previously described [29], 1 h after the pups’ separation from their dams to avoid interference between the rotavirus and milk components in the stomach. The studies were approved and conducted in accordance with the institutional guidelines for the care and use of laboratory animals as established by the Ethical Committee for Animal Experimentation of the UB and the Catalonia Government (CEEA-UB Ref.493/12, DAAM: 6905 for studies in rats and CEEA-UB Ref.494/12, DAAM: 6875 for the studies in mice for virus in vivo production).

### 2.2. Viruses

Two different type-A viruses have been used for the experiments: the simian agent 11 (SA11) and the epizootic-diarrhea infant-mouse virus (EDIM). The virus selected for the first infection in the model was the SA11, a rotavirus strain produced by the UB’s “Enteric Virus Group”, as in previous studies [23,26,29]. The virus selected for the second infection in the model was the EDIM strain, which is not able to grow in cell culture. Therefore, in order to obtain sufficient quantity of EDIM, an in vivo obtainment design was developed using an initial inoculum, which was a kind gift from Karen Knipping, Nutricia Research, Utrecht, The Netherlands. Neonatal BALB/c mice from 6 litters (*n* = 31) (Janvier) were inoculated at the age of 3 days with 5 μL of EDIM 0.9 × 10^8^ viral particles/mL. Stool samples were collected from day 4 to day 15 (corresponding to the days that the inoculated mice had diarrhea), pooled and homogenized using the Polytron^®^ (Kinematica, Luzern, Switzerland). EDIM was extracted with Genetron^®^ (1,1,2-trichloro-1,2,2-trifluoroethane, Sigma-Aldrich, Madrid, Spain) [34]. The quantification of EDIM particles was performed by ELISA (1.3 × 10^8^ viral particles/mL), as described in previous studies [34]. Its infectivity was later confirmed in mice: 5 µL of the new EDIM stock were inoculated to 3-day-old BALB/c mice from 3 litters (*n* = 15), causing diarrhea in all the animals (100% of incidence), between day 4 and 14 of life. Moreover, at the age of 21 days, splenocytes were isolated from some animals to test their specific proliferative response, which was significantly increased against RV particles. 

### 2.3. Experimental Designs

**Figure 1:**
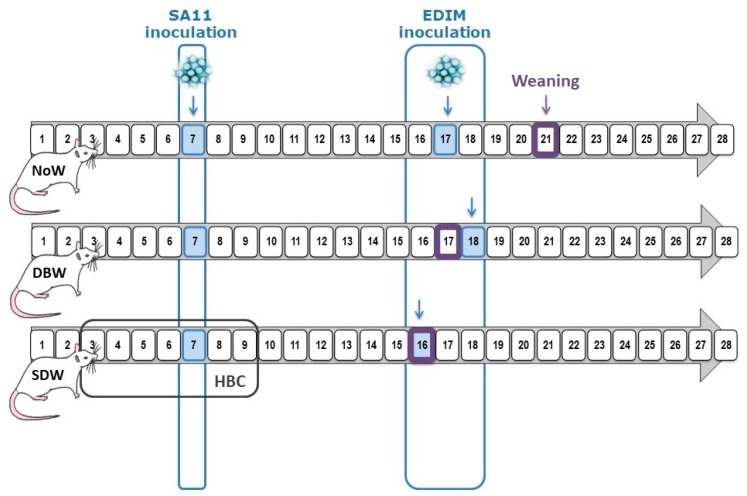
Experimental Design. Rotavirus inoculation days are marked in blue: SA11 inoculation was performed on day 7 and EDIM inoculation on days 16, 17 or 18. Weaning day is highlighted in a purple square (days 16, 17 or 21). These variables define the three experimental designs: normal weaning (NoW), same day weaning (SDW) and day before weaning (DBW). In addition, an anti-rotavirus hyperimmune bovine colostrum (HBC) was used as preventive agent in the SDW group.

Several experimental designs were utilized to identify the optimal conditions for the double rotavirus infection (DRI) model (Figure 1). SA11 was selected as a first infective virus because previous studies had shown that a rat model of mild diarrhea could be obtained using this strain of rotavirus in early life. EDIM was used as the second infective virus, and as no previous literature on the infectivity of EDIM in rats was found, a preliminary study was designed to confirm its infectivity in early-life rats (Table S1 and Figure S1). Moreover, the cross-reactivity against both types of viruses was also confirmed by means of ELISA and ELISPOT for anti- rotavirus Ab levels and secreting cells (SC) quantification in infected rats, respectively (Figure S2).

We established three critical points in the organization of these designs. Key criteria to consider were: the first infection was always performed in the first week of life (between day 6 and day 7), as our previous studies had demonstrated that no clinical signs are obtained later in life [29]; the second infection was induced early in the third week of life (between days 16 and 18) in order to try to induce infection before the intestinal immune system reached maturity [35]. Finally, conscious of the importance of the bioactive factors present in breast milk in protecting the pups from infection, the weaning day was either physiologically performed on day 21 or, in order to model a decreased immune function in neonates, was carried out on the same or the previous day of inoculation with the second infection. The designs’ nomenclature indicates the pattern of weaning with respect to the day of the second infection in the DRI groups of each design: normal weaning (NoW), same day weaning (SDW) and day before weaning (DBW). 

In all of them, the DRI groups of rats were inoculated with SA11 (~1.8 × 10^8^ TCID50 RV/rat in 100 μL of phosphate-buffered solution [PBS]) at day 6–7 of life. The second inoculation was induced with 100 μL of EDIM (~1.3 × 10^8^ rotavirus/mL) at days 16–18 of life in still suckling rats or early weaned (same or previous day) rats in order to obtain differential second-infection patterns. 

Each one of the experimental designs was composed of four different experimental groups: a non-inoculated reference (REF) group; a single-infection group infected with SA11 (SA11); a single-infection group infected with EDIM (EDIM); and a double-rotavirus infection (DRI) group infected with both SA11 first and later with EDIM (*n* = 2–4 dams/group with 6–9 pups each dam) (Figure 1). 

In addition to these experimental designs, an extra group as a dietary-like intervention was added to be used as a control group of protection during first infection. This group was constituted by DRI animals, which received “anti-rotavirus hyperimmune bovine colostrum” (HBC, kindly provided by Dr Viviana Parreño, Institute of Virology, CICV and A-INTA, Castelar, Argentina) in a concentration of 50 mg/animal/day in a 100 μL volume prior to and during first infection (from day 5 to day 13). This HBC was titrated to be effective in blocking the virus in vitro in concentrations higher than 10 μg/mL. This intervention would mimic a nutritional intervention with activity against viral infections such as breast milk components or some oligosaccharides [25,26].

Clinical evaluation was performed daily from the day before the first inoculation until the end of the study. Fecal samples were collected daily during the study and blood samples on day 28, when animals were euthanized (by cardiac puncture and exsanguination after ketamine/xylazine injection (Imalgene 100 mg/mL, Merial Laboratorios, Barcelona, Spain/Rompun^®^ 20 mg/mL, Bayer Hispania, Sant Joan Despí, Spain). Fecal pH and body temperature were measured after the infections. The delayed-type hypersensitivity (DTH) response and specific anti-rotavirus Ab in sera were determined at the end of the study.

### 2.4. Clinical Indexes and Faecal Specimen Collection

SA11 and EDIM infections were evaluated at 1 to 10 days post-inoculation (DPI) by the growth rate and clinical indexes that require daily fecal sampling, as previously described [29]. Fecal sampling was performed once a day by gently pressing and massaging the abdomen. Specimens were immediately scored in a blinded manner by 2–3 investigators for severity from 1 to 4 (diarrhea index (DI)), weighed and frozen at 20 °C for further analysis. The DI is based on colour, texture and amount as described: normal (1); loose yellow-green (2); totally loose yellow-green (3); high amount of watery (4) feces. Diarrhea scores >2 indicate diarrheic feces, whereas scores of DI <2 indicate the absence of diarrhea. Incidence of diarrhea was expressed by the percentage of diarrheic animals (%DA, consisting of the percentage of animals with diarrheic feces, taking into consideration the number of animals in each group) and by the percentage of diarrheic feces (%DF, consisting of the percentage of diarrheic samples, taking into consideration the number of total samples collected every day in each group).

### 2.5. Faecal pH and Body Temperature Determination

The pH from fecal diluted samples (up to 200 mg/mL in distilled water) from the peri-inoculation period was measured with a pH electrode for surfaces 5207 and a pH-meter micropH 2001 (Crison Instruments, Barcelona, Spain). Body temperature was measured with the TEMP JKT thermometer (Oakton, Vernon Hills, IL, USA) and the RET-3-ISO rectal probe for neonatal rats (Physitemp, Clifton, NJ, USA). This measure was taken during the peri-inoculation period of the virus. Results were expressed as the relative increase of temperature compared to the temperature the day before the inoculation taken as baseline.

### 2.6. ELISA for Specific Anti-Rotavirus Total Antibody Quantification in Serum and Viral Shedding

Ninety-six-well plates were coated with UV-inactivated SA11 particles at 10^5^/mL, and after blocking and incubating the sample (dilution 1/20), a rabbit peroxidase-conjugated anti-rat Ig (Dako, Barcelona, Spain) was used for detection, as in previous studies [29]. In the DBW design, sera anti-rotavirus IgM and intestinal anti-rotavirus Ig levels were also quantified as described [29]. Pooled sera from dams of inoculated litters were used as standard in each plate.

Fecal samples from 1–3 days after SA11 or EDIM inoculations were diluted in PBS (10 mg/mL) and homogenized using a FastPrep (MP Biomedicals, Santa Ana, CA, USA). Homogenates were centrifuged (13,000× *g*, 3 min), and supernatants were frozen at −20 °C until use. Rotavirus particles in fecal samples were quantified by ELISA, as previously described [29]. Titrated dilutions of inactivated SA-11 virus particles, ranging from 4 × 10^5^ to 2.5 × 10^4^/mL, were used as standard in each plate.

### 2.7. Delayed-Type Hypersensitivity

One day before sacrifice, the thickness of both the right and left ears of every animal were measured to constitute the basal values, using a pocket thickness gauge 7309 (Mituyoto, Hampshire, UK). For virus priming, animals were anaesthetized with isofluorane (Abbott Laboratories, Berkshire, UK) and a volume of 20 μL of UV-inactivated virus (~0.5 × 10^6^ rotavirus particles/mL) was injected into the right ear (RE) and the same volume of PBS was injected into the left ear (LE). After 24 h and prior to sacrifice, an evaluation of the ear thickness was performed again. Results are expressed as the increase of thickness (in mm) of the RE subtracting the increase of the thickness of the LE (in mm).

### 2.8. Statistical Analysis

The PASW Statistics 22 software package (SPSS Inc, Chicago, IL, USA) was used for the statistical analysis. The Komolgorov-Smirnov test was applied to assess normal distribution, followed by Levene’s test in order to determine variance equality. Conventional one-way ANOVA test was performed considering the experimental group as the independent variable. The results from different groups were sometimes pooled for a unique variable (e.g., SA11-infected rats from different groups vs. non-infected rats). When virus inoculation had a significant effect on the dependent variable, Scheffé’s post hoc test was applied. Kruskal-Wallis and Mann-Whitney U tests were used when non-normal distribution or different variances were found. Finally, the chi-square test was used to compare frequencies. Differences were considered significant at *p* values of <0.05. All the results are expressed as mean ± SEM of *n* animals.

### 2.9. Compliance with Ethical Standards

The studies were approved and conducted in accordance with the institutional guidelines for the care and use of laboratory animals as established by the Ethical Committee for Animal Experimentation of the UB and the Catalonia Government (CEEA-UB Ref.493/12, DAAM: 6905 for studies in rats and CEEA-UB Ref.494/12, DAAM: 6875 for the studies in mice for virus in vivo production).

## 3. Results

### 3.1. Clinical Variables and Body Weight during First Rotavirus Infection

**Figure 2:**
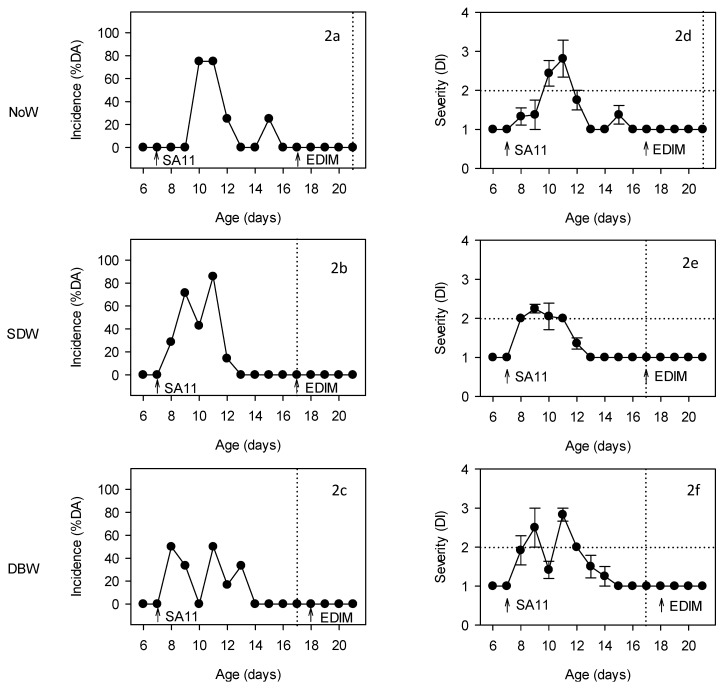
Evaluation of the incidence (**2a**–**2c**) and severity of diarrhea (**2d**–**2f**) of the DRI groups of the different experimental designs. Incidence is expressed as % of diarrheic animals (%DA) and severity as mean ± SEM of the diarrhea index (DI) (*n* = 6–12 animals/group). Arrows in each graph indicate the rotavirus inoculation day, first with SA11 and second with EDIM. Vertical dotted lines indicate the weaning day in each of the experimental designs. Horizontal dotted lines indicate the ID = 2, where higher values are indicative of diarrhea and lower values are indicative of no diarrhea.

First infection with SA11 during the first week of life induced diarrhea in all the inoculated groups in the three experimental designs conducted here, as can be observed from the incidence (%DA and %DF) and severity (DI) of diarrhea data. In Figure 2, only data from the DRI group of each design are represented, but results from the single-infected group (SA11 group) of each design followed a similar pattern to that in the DRI group. The %DA in the DRI group appeared from 1–3 DPI in all designs, reaching up to 50–100% between 1 and 4 DPI, and none of the animals in these designs still had diarrhea after 8 DPI. All designs showed a biphasic disease with two peaks of infection (Figure 2a–c) with and 70–100% of the SA11-infected animals developed diarrhea at some point. The results corresponding to the %DF followed a similar pattern as the %DA, achieving in all experimental designs a %DF of 100% at some point. None of the REF animals in any experimental design displayed diarrhea along the study, thus %DA or %DF was 0 and DI was 1 in all cases. Thus, in all designs the values of incidence and severity were statistically higher than those from the REF animals during the peaks of diarrhea: 3–4 DPI for NoW design, 2–4 DPI for SDW design and 1–2 and 4–5 DPI for DBW design (*p* < 0.05 vs. REF).

With regard to the severity of the diarrhea, the SA11-inoculated groups in all designs developed mild diarrhea (Figure 2d–f). The maximum diarrhea index ranged from 2.25–3 in all cases and was achieved around 2–4 DPI.

In all designs the diarrhea was also characterized by a fecal weight increase during the 4 days after infection when compared to REF animals, which showed no differences in any of the designs. The average weight of the fecal output during this period was 12.05 ± 1.71 g (mean ± SEM) in the SA11-infected groups from all designs, whereas in the REF group it was 4.05 ± 0.94 (*p* < 0.05). Finally, the viral infection and the consequent diarrhea did not significantly affect the growth curve of the animals in any of the three experimental designs, the overall body weight increase between day 4 and day 14 being about 190.44 ± 7.67 % in the REF groups and 177.82 ± 3.35 % in the SA11-infected groups.

### 3.2. HBC Protection during First Infection

**Figure 3:**
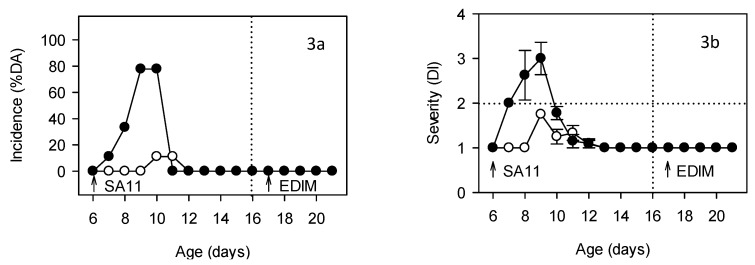
Evaluation of the incidence (**3a**) and severity of diarrhea (**3b**) of DRI animals (black circles) and DRI animals that received an “anti-rotavirus hyperimmune bovine colostrum” (HBC) supplement (white circles). Incidence is expressed as % of diarrheic animals (%DA) and severity as mean ± SEM of the diarrhea index (DI) (*n* = 9 animals/group). Arrows in each graph indicate the rotavirus inoculation day, first with SA11 and second with EDIM.

The intervention with the nutrional-like agent HBC when the pups were aged 5 to day 13 days old significantly reduced the incidence and severity of diarrhea. For instance, a maximum of 11% DA and 17% DF on days 4–5 DPI in the HBC group was achieved, whereas in SA11-infected animals, the values were of about 80% and 100%, respectively (Figure 3a). Moreover, the severity of the diarrhea of the few animals that developed it was also controlled, with a maximum diarrhea index of 1.75 ± 0.00 on 3 DPI (Figure 3b), a value that was lower than that of the SA11-infected rats (3.00 ± 0.36, *p* < 0.05). The onset of diarrhea was delayed by about two days (from 7 to 9 days old) and the duration of the disease was greatly shortened (the diarrhea period of the SA11 group was 1.67 ± 0.30 days and that of the HBC group was 0.22 ± 0.15 days). Fecal weight during the diarrhea period (day 7–10) was also significantly reduced in comparison with the SA11-infected animals, achieving values of 4.64 ± 0.99 g (*p* < 0.05 vs. SA11). Body weight was not affected as a result of the HBC intervention.

### 3.3. Clinical Variables and Body Weight after Second Rotavirus Infection

The second infection with EDIM during the third week of life failed to induce diarrhea in any of the inoculated groups—i.e., the single-(EDIM group) and the double-infection (DRI group) groups—in the three designs conducted here, as can be observed from the incidence and severity data (Figure 2).

Moreover, there were no differences in fecal weight among groups after the second infection. The absence of diarrhea after the EDIM inoculation, both in the single and in the double context, is paralleled with the absence of changes in the body weight gain during this period, being similar to those animals that did not receive any virus in this period (the body weight increase between day 6 and day 27 was 367.58 ± 6.54 % in the REF groups and 353.66 ± 9.18 % in the SA11 groups). Conversely, no physiological weight increase was evident for animals in the SDW and DBW groups during the 2–3 days after separation from their dams. As expected, due to the age-dependent insusceptibility to rotavirus, no clinical data evidenced the second infection with EDIM and therefore new variables were included in the study.

### 3.4. Faecal pH after Rotavirus Infections

The pH of fecal samples, as a possible marker of gastrointestinal alteration, was measured from 0–5 DPI after the onset of both rotavirus infections. Samples from SA11-infected animals (pooled results of SA11 and DRI groups of each design) showed a significantly lower fecal pH in two of the three designs during the acute diarrhea period compared to the REF group (Table 1). The fecal pH change due to the rotavirus infection was counteracted by the HBC-supplementation (5.26 ± 0.21, *p* > 0.05 vs. SA11 group). The fecal pH in the peri-infection period of the second infection with EDIM was also measured and, as expected due to the lack of changes in the fecal score, no differences were found between the infected groups and the REF group. 

### 3.5. Body Temperature after Rotavirus Infections

Rats’ body temperature in both SA11 and EDIM infections was monitored from 0–5 DPI as a possible marker of disease. The relative increase in temperature for the maximum value obtained after infection with respect to the 0 DPI value was calculated for each individual only in the weaned animal designs (SDW and DBW), because the temperature of suckling pups still with their mother (NoW) cannot be obtained (Table 2). After the first infection with SA11, an increase in rectal temperature was observed among the infected animals when compared to the REF group, but was only significant in the case of ED SDW (*p* < 0.05). Even though diarrhea was not observed in the second infection, an increase in body temperature was found in both the EDIM and the DRI groups (*p* < 0.05 vs. REF group in SDW and DBW designs), suggesting the presence of infection. On the other hand, the HBC oral supplementation in the SDW design did not have any positive effect on the temperature, either after the first infection (4.1 ± 0.2) or later in the second infection (4.4 ± 0.7). 

### 3.6. Viral Shedding after SA11 and EDIM Infections

In the DBW design, viral elimination was also studied, and in all rotavirus-inoculated animals, maximum viral shedding was observed on the first day after inoculation (1 DPI) with the SA11 infection. At that point no differential pattern as a result of HBC administration was observed, and the viral shedding of HBC group was 101.36 % of that of the DRI group. 

On day 18 (1 DPI for EDIM), the DRI group had lower viral shedding than those animals only infected with EDIM at day 17 (171.46% with respect to the DRI group’s viral shedding), but the difference was not statistically significant. However, in this case, the HBC-supplemented group showed 10 times higher viral clearance than the DRI group.

### 3.7. Anti-Rotavirus Antibodies Generated after SA11 and EDIM Infections

**Figure 4:**
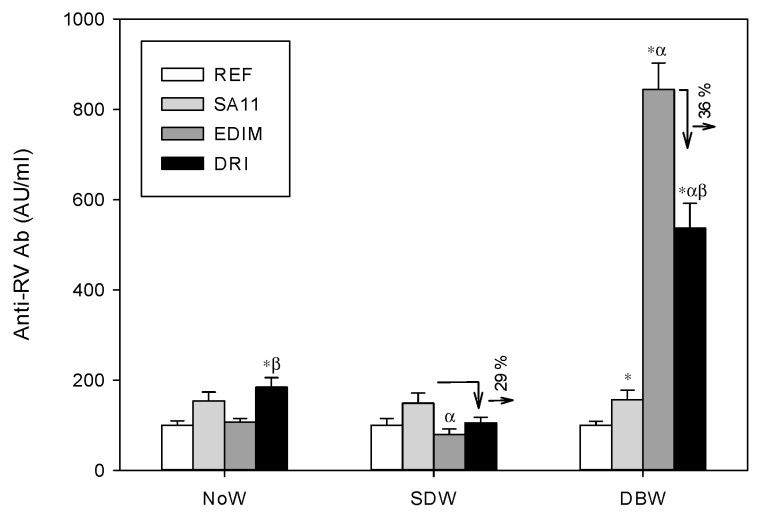
Specific anti-rotavirus total antibodies (Ab) in serum from 28-day-old rats from the different experimental designs (ED). Results are expressed as mean ± SEM (*n* = 6–12 animals/group) in AU/mL. Arrows indicate the percentage of reduction. Statistical differences: * *p* < 0.05 vs. REF group; ^α^ *p* < 0.05 vs. SA11 group; ^β^ *p* < 0.05 vs. EDIM group.

In all three designs the total serum anti-rotavirus specific Ab were studied in 28-day-old rats when their immune response was more mature (Figure 4). 

The anti-rotavirus Ab concentrations in SA11-infected groups were significantly increased by up to two-fold from those in the REF groups in all designs. The different conditions tested for the EDIM inoculation (i.e., continuing breastfeeding or early weaning the same day of infection) failed to induce a significant Ab response against the virus, with the exception of the DBW design (early weaning the day before infection), in which the anti-RV Ab concentrations increased more than eight times with respect to the REF group (*p* < 0.05).

When the immune response, after the combination of both viruses, was studied in the three different designs, two different patterns were found for the DRI with respect to a unique infection (either SA11 alone or EDIM alone): no effect (NoW) or a down-regulatory effect (SDW and DBW). Apart from the lack of effect in the NoW group (Ab response in the DRI group was similar to that in the SA11 group), the other two conditions generated an Ab response in animals from the DRI group, infected with both SA11 and EDIM, that was significantly lower than that when only SA11was inoculated (SDW) or EDIM (DBW), suggesting a down-regulatory effect of the second infective agent: a decrease of 29–36% in Ab levels was produced in these cases.

Anti-rotavirus IgM levels were also studied in sera obtained in the DBW design. Similarly, to the total anti-rotavirus Ig, there were higher titers of anti-rotavirus IgM in the EDIM group (905.03 ± 87.80 AU/mL) than in the REF (575.59 ± 28.71 AU/mL) and SA11 (859.30 ± 304.37 AU/mL) groups (*p* < 0.05). Moreover, the pattern in the DRI was different to those of the single infection groups (711.26 ± 95.59 AU/mL). 

In addition, in the DBW model, a similar pattern was found for intestinal anti-rotavirus Ig in small intestinal gut washes: higher levels were found in the SA11 and EDIM single infections but not in the DRI group compared to the REF group. Regarding the nutritional intervention, there was no evidence that the HBC protection during the first infection decreased the serum anti-rotavirus Ig and anti-rotavirus IgM titers in the DRI group with respect to the single infections, but it was able to increase 1.5 times the anti-rotavirus Ig levels in the intestinal compartment with respect to the DRI group (*p* < 0.05). 

### 3.8. Delayed Type Hypersensitivity

**Figure 5:**
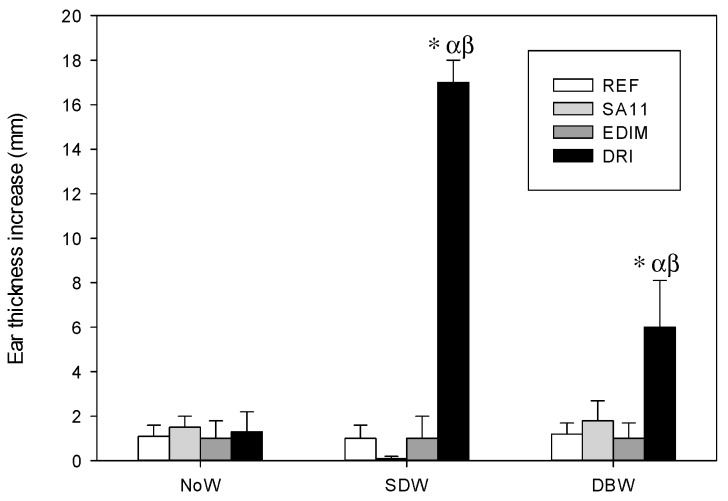
Delayed-type hypersensitivity (DTH) response in some of the experimental designs (ED) in 28-day-old animals. Results are expressed as mean ± SEM of the 24 h-increase of thickness of the rotavirus-injected ear subtracting the increase of thickness of the PBS-injected ear (*n* = 6–12 animals/group). Statistical differences: * *p* < 0.05 vs. REF group; ^α^ *p* < 0.05 vs. SA11 group; ^β^ *p* < 0.05 vs. EDIM group.

The DTH response at 24 h was studied in all three designs, showing a clear up-regulatory effect of double infection in the early weaned animals (Figure 5). The groups infected with SA11 and EDIM alone did not show an increase in this response when compared to the REF group, but the DTH response increased in the DRI group in the design involving early weaning, either on the same day of infection (SDW) or the day before (DBW) (*p* < 0.05). The nutritional intervention with HBC was able to significantly reduce such an increase in the SDW design, drastically reducing the ear thickness of the ear to REF levels.

## 4. Discussion

Group A rotavrius is the most common cause of acute diarrhea among children under the age of five worldwide [36,37]. Due to its impact on the health system and society, it is of interest to explore the rotavirus infection and its pathology, along with the clinical and immune response, and to test its possible modulation by nutritional compounds. Several rotavirus-infection animal models are already available, but most of them are single-infection models, which do not reflect the multiple reinfections that humans have during early life. Hence, the purpose of this study was to develop a double-infection model in rat, which is more similar to clinical reality than a single-infection model and to study the preventive scenario, such as a dietary intervention, for providing immunity already during first infection. 

First, rats from the Lewis strain were selected, as these have a high susceptibility to infection by rotavirus whereas the Wistar strain has mechanisms to avoid virus replication [29]. Age is also an important factor, because rodents can be infected at any age, but they do not always develop the disease. In particular, rats only present clinical signs when they are infected before 21–27 days of life, and the occurrence of diarrhea is inversely age-dependent [28,29,37].

Two rotavirus inoculations were performed in the different experimental designs tested in the present study. The first one, with SA11, was at the end of the first week of life, in line with previous studies [26,29]. A 5-day mild diarrhea was achieved, without body weight loss, but with fecal weight increase in the acute diarrhea period, as in previous studies [26,29]. In other animal models with a more severe diarrhea disease, such as in mice, body weight loss has been described [38]. For the second infection, mimicking reinfections that often occur in infants, the rotavirus EDIM was chosen and inoculated at day 17 of life, as previously established in mice [34], confirming its infectivity on rats. In this case, diarrhea was not observed due to the older age of the animals, as in the other models of rotavirus double-infection in other species [30,31,32,33,34]. To enhance the rotavirus infection, the inoculation was performed on day ~17 of life in the different experimental designs, but with early weaning introduced on the same day, or even one day before, which imposes a physiological stress on the animal. This change in the weaning day still did not provoke the apparition of clinical features, and therefore other markers needed to be assessed.

The fecal pH seems to decrease with a rotavirus infection in two of the three designs studied, but only when diarrhea is obtained (first infection). This may be because of the body’s electrolyte imbalances caused by the diarrhea [13]. However, Li et al. [39] observed an increase in pH in the colonic content of rotavirus-infected piglets. In our case, measuring pH did not aid in the assessment of the second infection. We confirmed a temperature rise after both the first infection—but only in one of the two designs in which this variable was studied—and the second infection, even though no clinical signs were observed in the latter. However, no different pattern was observed between the single and the double infection. Few studies have evaluated the body temperature. For example, although Parreño et al. [33] measured temperature after rotavirus infection in a calf model and observed fever, they only measured it after the first infection, not after the second. Overall, and regardless of the fact that the results in our study were not conclusive in all the designs, pH and temperature measures might be useful as disease indicators in these types of models, although the results are more evident in the first infection. A current limitation of this study that must be taken into consideration in the future is that we did not have a technique with enough sensitivity to be able to evaluate the viral shedding in all the experimental designs; a tool used in previous studies [23,26] and by others could have allowed us to observe the impact of the first infection on the prevention of the second one.

Finally, changes in immune response were assessed in order to obtain differential patterns in the double infection in terms of specific Ab and DTH response. These variables were determined at the end of the study, when the immune system of the animals was more developed. The selection of these variables was performed according to the guidelines published by Albers et al., in 2013 [27].

The DTH response after second infection was found to be up-regulated by a first infection in the early weaned designs. No DTH response was found in the first design (NoW), probably due to the breast milk components that participate in the protection against RV during both infections but especially in the second one and therefore highlighting the importance of infant’s nutrition and breastfeeding in this context. On the contrary, in the SDW and the DBW designs, the single infections did not induce DTH response but a clear effect was observed in the DRI animals. These results are contrary to what has been observed in other studies, where the DTH response was detected after an infection at day 17 in mice, but was suppressed after a reinfection in mice who had previously received a primary infection [34,40]. Our results may suggest that animals that have already been in contact with the virus (first infection), are able to respond more strongly to the DTH stimuli after the second infection. This result is supported by the fact that it is known that rotavirus-specific CD8+ T cells developed after a first infection mediate the resolution of the second infection [19,41]. 

In the SA11 groups of all the designs, increased levels of Ab compared to the REF groups were already found, similarly to previous studies [29,30,32]. With regard to the second infection, the results from the NoW (physiological weaning) design suggest that the second infection was not strong enough to modify the Ab levels (those from the DRI group were similar to those from the SA11 group), but when an early weaning was performed, the second infection regulated this variable (SDW design) and did so more significantly if the animals had been weaned one day before the EDIM inoculation (DBW design). Thus, a down-regulatory action was found on the humoral response in the second infection after the first one. This has also been shown by Sheridan et al., in a model of reinfection in adult mice [40]. In contrast with these results, Knipping et al., did not find a differential Ab pattern between single and double infections [34]. Overall, the low titers of anti-rotavirus Ab in the DRI animals may be linked to the higher cell response achieved after first infection, which could protect against the virus without needing to highly activate the humoral immune response. However, it has to be highlighted that levels of Ab in the breast milk are variable and can confer protection to the pups, presumably due to the previous contact of the dams with rotavirus in their respective animal facilities. Even though we agree that despite the importance of evaluating anti-rotavirus Ab titers in pups’ sera, it is also important to determine those of the dams before starting an interventional study with this type of model and, if possible, stratifying the groups by high vs. low dam maternal rotavirus antibody titers.

Moreover, the HBC is an effective protection in the control of diarrhea in this and other rotavirus models. In other studies, the administration of Gastrogard-R^®^ (colostrum containing rotavirus specific Ab) also protected suckling mice against the first rotavirus infection, but the fecal viral load after the second infection was as high as the first one would be, although it allowed the development of B and T cell responses [34]. Parreño et al., described full protection by HBC after a first and second virus exposure in calves [33]. A combination of HBC and the probiotic strain *Lactobacillus rhamnosus* GG also protected mice successfully from rotavirus diarrhea [42]. Other authors have also found diarrhea protection or a reduction of rotaviral disease in mice with the administration of bovine colostrum from healthy cows [43,44] or whey protein concentrates [23,25,45], sometimes with diminished Ab titers [23] and sometimes with no differences among groups [45]. In the case of multiple infections, HBC intervention may help in understanding the influence of regulatory interventions by nutrients during these processes. In our case, the protection from the first infection by HBC leads to the induction of a lower immune response after the second infection (in terms of Ab and DTH response), suggesting that HBC, in the first rotavirus infection, is able to effectively block the virus, promote its elimination and therefore diminish its infectivity and disease but also the priming of immunity in this sense. Future studies should be directed to defining an intermediate dose or to finding protective agents that may control disease but also allow the development of own immunity against the pathogen.

## 5. Conclusions

In summary, the early weaning and an early age are important features for the model to achieve a valid design candidate for both infections. The model includes clinical signs during first infection, but as expected, not in the second, and regulation of immune response due to the first rotavirus contact. The diarrhea index and the fecal pH are suitable tools for assessing the first infection, body temperature is an appropriate clinical variable for both infections, and specific Ab in serum at day 28 and the DTH response are useful variables for evaluating the second rotavirus infection. We can conclude that the rotavirus double-infection rat model is suitable for studying the influence of interventions performed to regulate first infections (e.g., by vaccination, therapeutic agents and especially nutritional supplementation) on the onset of a future reinfection, which so often occurs in humans.

## Figures and Tables

**Table 1:** Fecal pH in the peri-inoculation period (first infection). Results are expressed as mean ± SEM (*n* = 4–8 samples/group).

	1st Infection	
	REF	SA11
**NoW**	4.83 ± 0.05	4.56 ± 0.03 *
**SDW**	5.07 ± 0.16	4.48 ± 0.07 *
**DBW**	4.36 ± 0.05	4.76 ± 0.21

* *p* < 0.05 vs. REF group.

**Table 2:** Rectal temperature of the animals in the peri-inoculation period (first and second infection). Results are expressed as mean ± SEM (*n* = 6–12 animals/group) of the relative increase of temperature with respect to the temperature the day before virus inoculation (basal value).

	1st Infection (2 DPI)		2nd Infection (1 DPI)		
	REF	SA11	REF	EDIM	DRI
**SDW**	0.47 ± 0.32	4.35 ± 1.22 *	0.12 ± 0.12	4.40 ± 0.77 *	3.60 ± 0.42 *
**DBW**	0.40 ± 0.32	0.83 ± 0.46	3.24 ± 0.81	5.53 ± 0.38 *	7.50 ± 0.72 *

* *p* < 0.05 vs. REF group.

## Supplementary Materials

The following are available online at http://www.mdpi.com/2072-6643/11/1/131/s1, Table S1: Evaluation of the incidence and severity of diarrhea when EDIM was inoculated at day 3 or day 6 of life, Figure S1: Proliferative response against RV on 21-day-old rats that were infected with EDIM at day 3 of life, Figure S2: SA11 and EDIM cross-reactivity.
