# Supplementary material for: Rotavirus Double Infection Model to Study Preventive Dietary Interventions
Source: Nutrients. 2019 Jan 10;11(1):131. doi: 10.3390/nu11010131 (PMC6357201; doi:10.3390/nu11010131)
Supplement: Supplementary file 1 [file nutrients-11-00131-s001.pdf]

nutrients

MDPI

SUPPORTING INFORMATION

Table S1. Evaluation of the incidence and severity of diarrhea when EDIM was inoculated at day 3 or day 6 of life. One litter of 3-day-old rats (EDIM d3 group, n=8) was inoculated with $1.3x10^5$ viral particles/animal and another litter of 6-day-old rats (EDIM d6 group, n=8) was inoculated with $2x10^6$ viral particles/animal of the EDIM aliquot generated *in vivo* in mice. A differential dose was used at different ages due to the age-dependent resistance to infection of suckling rats. In both cases, there was a low but significant incidence of diarrhoea during the following days after the virus inoculation, which in any case was higher than 50 % of diarrhoeic animals (%DA). The mean severity of the inoculated animals did not achieve values ≥ 2, overall meaning faeces with not normal appearance (loose, watery, trend to yellow-green) but with no high diarrhoeic features. This pattern was extended from the day after virus infection (1 DPI) to at least 4–5 days later, with the effect being more pronounced the earlier the infection was introduced (d3 vs. d6), even though the virus dose was higher in the older animals. This mild diarrhoea did not induce any body weight loss (data not shown).

	**EDIM d3**		**EDIM d6**	
	**DI**	**%DA**	**DI**	**%DA**
**d3**	1.00 ± 0.00	0.00	1.00 ± 0.00	0.00
**d4**	1.50 ± 0.29	22.22	1.00 ± 0.00	0.00
**d5**	1.50 ± 0.14	11.11	1.00 ± 0.00	0.00
**d6**	1.25 ± 0.25	0.00	1.50 ± 0.22	33.33
**d7**	1.08 ± 0.08	0.00	1.38 ± 0.24	16.67
**d8**	1.21 ± 0.10	0.00	1.71 ± 0.16	50.00
**d9**	1.11 ± 0.04	0.00	1.35 ± 0.22	16.67
**d10**	1.75 ± 0.09	33.33	1.00 ± 0.00	0.00
**d11**	1.38 ± 0.13	0.00	1.00 ± 0.00	0.00

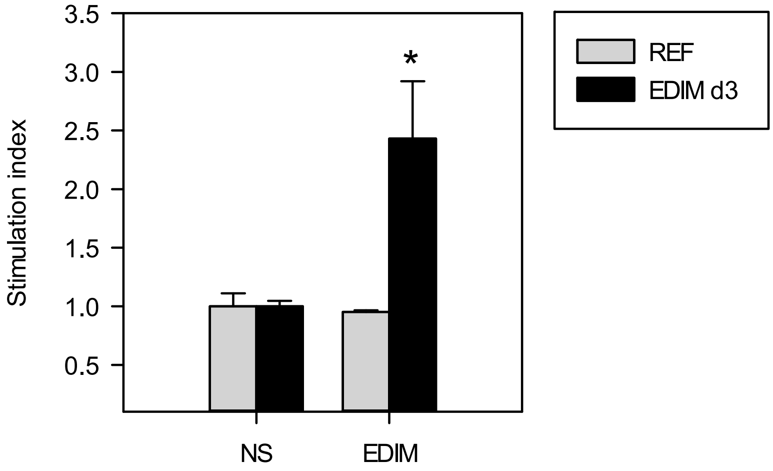

Figure S1. Proliferative response against RV on 21-day-old rats that were infected with EDIM at day 3 of life. Regarding the immune response at the end of the study, there was a higher proliferative response in splenocytes from EDIM d3 rats when stimulated with the RV than in those from the REF group, indicating that EDIM was able to infect and prime the neonatal rat immune system. However, no increase was found in EDIM d3 and EDIM d6 serum anti-RV antibodies with respect to REF animals (data not shown). NS, non-stimulated cells; EDIM, cells stimulated with 10^4 particles EDIM/well. Proliferating cells were identified by means of BrdU Cell Proliferation Assay Kit (Merck Millipore, Darmstadt, Germany), following manufacturer's instructions. Results are expressed as mean ± S.E.M. Statistical difference: *$p<0.05$ vs. NS.

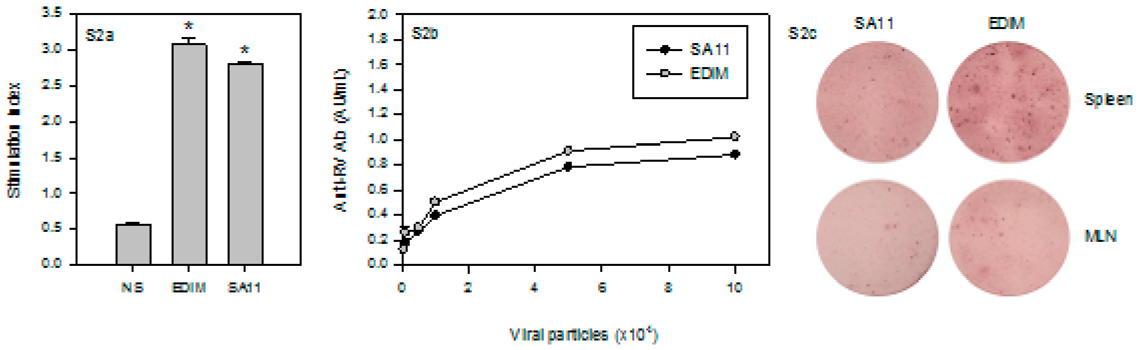

Figure S2. SA11 and EDIM cross-reactivity. In order to ensure that a first infection with SA11 would be able to modulate a subsequent infection with EDIM, the cross-reactivity among both viruses was studied. **(S2a) Proliferative response against different RV stimulus in rats infected with SA11.** The specific proliferative capacity under diverse RV stimuli (SA11 or EDIM) was studied in SA11-infected rat splenocytes. Cells were able to proliferate in both conditions, achieving a significant three-fold increase of proliferation when compared with non-stimulating conditions ($p<0.05$). NS, non-stimulated cells; EDIM, stimulated with 10^4 EDIM particles/well; SA11, stimulated with 10^4 SA11 particles/well. Results are expressed as mean ± SEM. Statistical differences: *$p<0.05$ vs. NS. **(S2b) Quantification of antibody titres from SA11-infected rats in SA11 or EDIM-coated ELISA wells at different concentrations.** To test the specificity of serum anti-RV Ab developed by SA-11-infected animals, their cross-reactivity was analysed by ELISA. There was an increasing positive signal depending on the amount of virus coated to the plate but it did not depend on the type of virus (SA11 or EDIM). The increasing signal of the Ab present in the serum was dependent on the amount of virus coated to the well and therefore is dose-dependent and, indeed, has a similar pattern between both types of virus. Results are expressed as mean ± SEM. **(S2c) Representative images of detection by ELISPOT of spontaneous anti-RV IgM-SC either from the spleen or the MLN of SA11-infected rats.** Specific anti-RV Ab-SC from SA11-infected animal spleen or MLN were detected by the ELISPOT technique. Anti-RV IgM-SC were clearly detected – with more signal in the spleen than in the MLN cells – against both SA11 and to EDIM, showing the capacity of SA11-sensitized animals to also recognize EDIM. 200.000 cells plated in each well, SA11 or EDIM at the top indicate the coating antigen (10^5 particles/mL) used in each nitrocellulose well. Conditions used as in previous publications of the group.
